# An Antitubulin Agent BCFMT Inhibits Proliferation of Cancer Cells and Induces Cell Death by Inhibiting Microtubule Dynamics

**DOI:** 10.1371/journal.pone.0044311

**Published:** 2012-08-31

**Authors:** Ankit Rai, Avadhesha Surolia, Dulal Panda

**Affiliations:** 1 Department of Biosciences and Bioengineering, Indian Institute of Technology Bombay, Mumbai, Maharashtra, India; 2 Molecular Biophysics Unit, Indian Institute of Science, Bangalore, Karnataka, India; Semmelweis University, Hungary

## Abstract

Using cell based screening assay, we identified a novel anti-tubulin agent (Z)-5-((5-(4-bromo-3-chlorophenyl)furan-2-yl)methylene)-2-thioxothiazolidin-4-one (BCFMT) that inhibited proliferation of human cervical carcinoma (HeLa) (IC_50_, 7.2±1.8 µM), human breast adenocarcinoma (MCF-7) (IC_50_, 10.0±0.5 µM), highly metastatic breast adenocarcinoma (MDA-MB-231) (IC_50_, 6.0±1 µM), cisplatin-resistant human ovarian carcinoma (A2780-cis) (IC_50_, 5.8±0.3 µM) and multi-drug resistant mouse mammary tumor (EMT6/AR1) (IC_50_, 6.5±1****µM) cells. Using several complimentary strategies, BCFMT was found to inhibit cancer cell proliferation at G2/M phase of the cell cycle apparently by targeting microtubules. In addition, BCFMT strongly suppressed the dynamics of individual microtubules in live MCF-7 cells. At its half maximal proliferation inhibitory concentration (10 µM), BCFMT reduced the rates of growing and shortening phases of microtubules in MCF-7 cells by 37 and 40%, respectively. Further, it increased the time microtubules spent in the pause (neither growing nor shortening detectably) state by 135% and reduced the dynamicity (dimer exchange per unit time) of microtubules by 70%. *In vitro*, BCFMT bound to tubulin with a dissociation constant of 8.3±1.8 µM, inhibited tubulin assembly and suppressed GTPase activity of microtubules. BCFMT competitively inhibited the binding of BODIPY FL-vinblastine to tubulin with an inhibitory concentration (K_i_) of 5.2±1.5 µM suggesting that it binds to tubulin at the vinblastine site. In cultured cells, BCFMT-treatment depolymerized interphase microtubules, perturbed the spindle organization and accumulated checkpoint proteins (BubR1 and Mad2) at the kinetochores. BCFMT-treated MCF-7 cells showed enhanced nuclear accumulation of p53 and its downstream p21, which consequently activated apoptosis in these cells. The results suggested that BCFMT inhibits proliferation of several types of cancer cells including drug resistance cells by suppressing microtubule dynamics and indicated that the compound may have chemotherapeutic potential.

## Introduction

The development of resistance to the existing anticancer drugs and tumor metastasis are among the major obstacles in cancer chemotherapy [1], [2]. Cell based cytotoxicity assays have been found to be an attractive screening method to discover anticancer agents. Several of the clinically effective anticancer agents were first identified through high throughput screening [3]–[6]. For example, taxol was first discovered through a high throughput cell based screening assay [6].

Microtubules are structural components of the mitotic spindle and the dynamic microtubules play an important role in several cellular processes including intracellular trafficking, cell migration and cell division [7]. Several of the mitotic inhibitors are known to inhibit microtubule assembly [8]–[12] and the inhibition of microtubule assembly dynamics has been shown to be the mode of action for several clinically successful anticancer drugs including vinblastine, vincristine, estramustine and paclitaxel [11], [12]. In addition to their clinical applications in diverse types of diseases including cancer, fungal and parasitic diseases [13], microtubule inhibitors are also highly useful for understanding the role of microtubules in the cellular processes [14], [15]. Microtubule inhibitors generally block cell cycle progression in mitosis and a prolonged mitotic-arrest triggers various apoptotic pathways [12], [16], [17].

Rhodanine derived compounds are acquiring attention in chemotherapy because of the presence of heterocyclic ring (2-thioxothiazolidin-4-one) in the parent scaffolds [18]. Substitutions on the heterocyclic ring provide an excellent opportunity to formulate novel derivatives with broad range of biological activities [18]. Biological activities of rhodanine derived compounds have been examined in various studies [18] and these agents exhibited antibacterial [19], [20], antimalarial [21], antiviral [22] and anticancer potential [23]. In the present work, we aimed to find a new chemical entity having antiproliferative and antimitotic activities from a large subset (a library of 156 compounds) of rhodanine derived scaffolds with an idea that the compound may have anticancer potential.

We found that three compounds namely (E)-5-((5-(2-methyl-5-nitrophenyl) furan-2-yl)methylene)-2-thioxothiazolidin-4-one (MNFMT), (E)-5-(3,5-dichloro-4-hydroxybenzylidene)-3-phenyl-2-thioxothiazolidin-4-one (DHBPT) and (Z)-5-((5-(4-bromo-3-chlorophenyl) furan-2-yl) methylene)-2-thioxothiazolidin-4-one (BCFMT) inhibited the proliferation of HeLa and MCF-7 cells in culture. Among these compounds, BCFMT was found to increase the mitotic cell population of HeLa and MCF-7 cells more potently than the other two compounds. BCFMT inhibited the assembly of purified tubulin *in vitro* and in cultured cells while MNFMT and DHBPT did not show any significant effect on tubulin assembly. We obtained several lines of evidence suggesting that BCFMT exerts its antiproliferative and antimitotic activities by dampening dynamic instability of individual microtubules in cultured cells through binding at the vinblastine site in tubulin. In addition, BCFMT potently inhibited the proliferation of drug resistant namely cisplatin resistant human ovarian carcinoma A2780-cis and multi-drug resistant mouse mammary tumor EMT6/AR1 cells and highly metastatic MDA-MB-231 cells suggesting that it may have chemotherapeutic potential.

## Materials and Methods

### Materials

Sulforhodamine B, bovine serum albumin, mouse anti-α-tubulin IgG, mouse anti-β-actin IgG, FITC conjugated anti-rabbit IgG were obtained from Sigma, St. Louis, MO, USA. Alexa fluor 568 goat anti-mouse IgG was purchased from Molecular Probes, Invitrogen, CA, USA. Mouse anti-cyclin B1, rabbit anti-p-Histone H3 (Ser 10), mouse anti-p53 IgG, mouse anti-p21 IgG antibodies and apoptosis detection Kit (Annexin V-Propidium Iodide) were purchased from Santa Cruz Biotechnology, CA, USA. Mouse anti-BubR1 IgG was obtained from BD Biosciences, CA, USA. Rabbit anti-Mad2 IgG was purchased from Bethyl laboratories, Montgomery, USA. Mouse anti-Hec1 IgG was purchased from Abcam, Cambridge, MA, USA. Fetal bovine serum was obtained from Biowest, Nuaille, France. All other reagents were of analytical grade and obtained from Sigma, MO, USA and Himedia, Mumbai, India. All compounds tested were obtained from Chembridge Corporation, San Diego, CA, USA.

### Cell Culture

Human cervical carcinoma (HeLa), human breast adenocarcinoma (MCF-7) and metastatic breast adenocarcinoma (MDA-MB-231) cells were obtained from cell repository of National Centre for Cell Science, (NCCS) Pune, India. NCCS characterized the cells by mt-rDNA sequence to confirm the species. These cell lines were found to be free of mycoplasma. Cisplatin-resistant human ovarian carcinoma (A2780-cis) cells and multi-drug resistant mouse mammary tumor (EMT6/AR1) cells were purchased from Sigma, St. Louis, MO, USA. Cell line authentication was done by short tandem repeat profiling and isoenzyme analysis by the supplier and was also reported negative for the presence of mycoplasma.

HeLa and MCF-7 cells were cultured in Eagle’s Minimal Essential Medium (MEM). MDA-MB-231 cells were grown in Leibovitz's L-15 Medium. A2780-cis cells were maintained in RPMI-1640 media containing 1 µM cisplatin. EMT6/AR1 cells were grown in MEM medium containing 1 µg/ml doxorubicin. Media were supplemented with 10% fetal bovine serum, 2.2 g/l sodium bicarbonate and 1% antibiotic-antimycotic solution containing streptomycin, amphotericin B and penicillin. Cells were grown and maintained at 37°C incubator in humidified atmosphere of 5% CO_2_ and 95% air.

### Screening for Antiproliferative Activity of Rhodanine Series of Compounds

The antiproliferative potential of 156 rhodanine derived compounds against HeLa cells was determined by sulforhodamine B assay [24], [25]. HeLa cells (1**×**10^5^ cells/ml) were seeded in 96-well cell culture plates. Stocks of compounds were prepared in DMSO. After 24 h of seeding, the media was replaced with fresh media containing either vehicle (0.1% DMSO) or 2 µM of each of the rhodanine compounds. After 24 h of incubation with different compounds, cells were fixed with 10% TCA and processed for sulforhodamine B assay [24], [25].

To determine the half maximal inhibitory concentration (IC_50_) of MNFMT, DHBPT and BCFMT, 1**×**10^5^ cells/ml HeLa and MCF-7 cells were seeded in 96 well cell culture plates. Different concentrations of compounds were diluted in media and added in the wells after 24 h of cell seeding. HeLa and MCF-7 cells were grown in the absence and presence of compounds for 24 h and 48 h, respectively. Inhibition of cell proliferation in the presence of compounds was determined using standard sulforhodamine B assay. Data were an average of three independent experiments.

### Light Scattering Experiment

The effects of MNFMT, DHBPT or BCFMT on the assembly of purified tubulin were monitored by light scattering at 400 nm. Tubulin was purified as described previously [26], [27]. Tubulin (10 µM) in PEM buffer (25 mM PIPES pH 6.8, 3 mM MgCl_2_, 1 mM EGTA) and 1 M glutamate was incubated without and with different concentrations of MNFMT, DHBPT or BCFMT on ice for 10 min. Then, 1 mM GTP was added to the reaction mixtures and the assembly kinetics was monitored at 37°C using a FP-6500 spectrofluorometer JASCO, Tokyo, Japan.

### Electron Microscopy

Tubulin (10 µM) was polymerized without or with 25 and 50 µM BCFMT at 37°C for 15 min as described above. The samples were fixed with 0.5% glutaraldehyde, transferred to carbon-formvar coated grids (Electron Microscopy Sciences, USA) and negatively stained with 2% uranyl acetate. Samples were visualized under electron microscope (Tecnai G^2^12, FEI, Eindhoven, Netherlands).

### Effects of BCFMT on the GTPase Activity of Microtubules in vitro

Tubulin (25 µM) in 4 M glycerol, 5 mM MgCl_2_ and 1 mM GTP was polymerized at 37°C for 30 min. Microtubule seeds were generated by shearing the polymers through a 23 gauge needle of a 5 ml syringe. Tubulin (15 µM) in PEM buffer and 1 mM GTP was polymerized by adding 20% (v/v) microtubule seeds at 37°C for 10 min. After 10 min of polymerization, different concentrations of BCFMT were added into the reaction mixtures and further polymerized for 30 min. The hydrolysis reaction was stopped by adding 10% (v/v) of 7 M perchloric acid and the amount of inorganic phosphate released was determined by malachite green assay [28].

### Measurement of the Dissociation Constant of the Binding of BCFMT to Tubulin Using Tryptophan Fluorescence of Tubulin

Tubulin (2 µM) in 25 mM PIPES buffer pH 6.8 was incubated without and with different concentrations of BCFMT at 25°C for 20 min. The fluorescence intensity was monitored by exciting the reaction mixture at 295 nm and the emission spectrum was recorded in the range of 310 nm to 370 nm. A fluorescence cell of 0.3 cm path length was used and the fluorescence intensities were corrected for inner filter effect using the formula
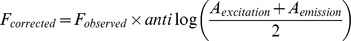



Fluorescence data were fitted in the following equation;
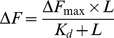
Where, ΔF is the change in the fluorescence intensity of tubulin in the presence of BCFMT, ΔF_max_ is the maximum change in the fluorescence intensity of tubulin when it is saturated with BCFMT and C is the concentration of BCFMT. The dissociation constant (K_d_) for BCFMT binding to tubulin was estimated using the Graph Pad Prism 5 software (Graph Pad Software, CA, USA).

### Effect of BCFMT on BODIPY FL-vinblastine Binding to Tubulin

Tubulin (2 µM) in 25 mM PIPES buffer pH 6.8 was incubated without and with 10, 25 and 50 µM BCFMT for 20 min at 25°C. BODIPY FL-vinblastine (2 µM) was added in the reaction mixtures and incubated at 25°C for an additional 20 min in dark. Tubulin-BODIPY FL-vinblastine complex was excited at 490 nm and the emission spectrum was taken in the range of 500–550 nm [29]. The spectrum of BODIPY FL-vinblastine in the absence of tubulin was also monitored.

### Immunofluorescence Microscopy

MCF-7 or HeLa cells were seeded at a density of 5**×**10^4^ cells/ml on polylysine-coated glass coverslip in 24-well cell culture plate. After 24 h of seeding, different concentrations of BCFMT were added in the wells. Control cells were treated with vehicle (0.1% DMSO). After 24 or 48 h of incubation with BCFMT, immunostaining was performed using antibody against α-tubulin, p53, p21, BubR1, cyclin B1, β-actin (1° 1∶300, 2° 1∶300 alexa-568 labeled), phosphohistone–H3 (Ser 10) (1° 1∶300, 2° 1∶300 FITC labeled), Hec 1 (1° 1∶800, 2° 1∶800 alexa-568 labeled) and Mad2 (1° 1∶500, 2° 1∶500 FITC labeled) as described earlier [29]–[32]. DNA was stained with Hoechst 33258 (1 µg/ml). Images were taken using Eclipse TE 2000U microscope (Nikon, Tokyo, Japan) at 40× magnification and processed using Image-Pro Plus software (Media Cybernetics, Silver Spring, MD).

### Measurements of Microtubule Dynamics in MCF-7 Cells

Transfection of EGFP-α tubulin construct in MCF-7 cells was done using lipofectamine-2000 [29]. The kinetic parameters for the dynamic instability of microtubules were determined as described earlier [8], [31]–[34].

### Western Blot Analysis

MCF-7 cells were incubated in the absence and presence of 20 and 40 µM of BCFMT for 36 h. The effect on the polymerized amount of microtubules in the cells was determined by western blotting using monoclonal antibody for α-tubulin as described previously [31]. Intensity of the protein bands was calculated using Image J software version 1.43u.

### Effects of BCFMT on the Kinetics of the Release of Nocodazole-induced Mitotic Block in MCF-7 Cells

MCF-7 cells were blocked in M phase of cell cycle after 24 h of treatment with 1 µM nocodazole. To remove nocodazole, cells were cytospinned and washed carefully three times with fresh media. After nocodazole removal, cells were incubated in the absence and presence of 40 µM BCFMT at 37°C. Cells were fixed at 0, 1, 2 and 4 h of incubation with BCFMT at 37°C incubator. Fixed cells were stained with Hoechst 33258 and the mitotic cells were scored (n = 3; in each set 1000 cells were counted).

### Annexin V/PI Staining

MCF-7 cells (5**×**10^4^ cells/ml) were seeded on polylysine-coated glass coverslip in a 24-well cell culture plate. After 24 hours of seeding, cells were incubated without and with different concentrations of BCFMT for 48 hours. Cells were collected by cytospinning at 2400 rpm at 30°C for 10 min. Annexin V/PI staining was performed as described earlier [30].

## Results

### Effects of Rhodanine Derivatives on the Proliferation of HeLa and MCF-7 Cells

**Figure 1:**
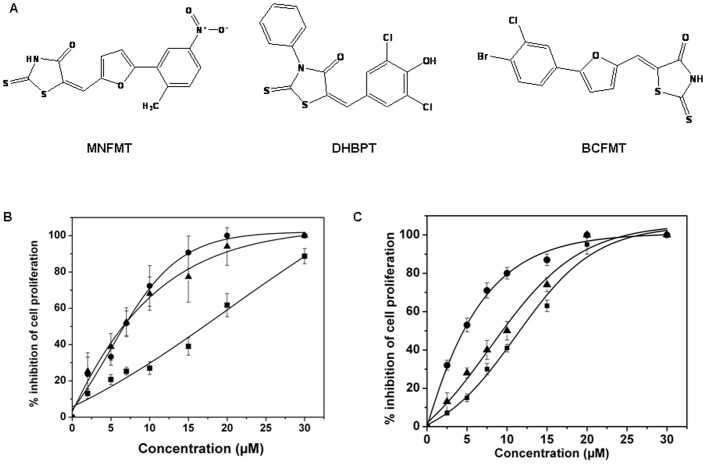
Effects of MNFMT, DHBPT and BCFMT on HeLa and MCF-7 cells proliferation. (A) Structures of MNFMT, DHBPT and BCFMT. (B & C) MNFMT, DHBPT and BCFMT inhibited the proliferation of HeLa (B) and MCF-7 (C) cells in culture. HeLa and MCF-7 cells were incubated with different concentrations of MNFMT (▪), DHBPT (•) and BCFMT (▴) for one cell cycle. The inhibition of cell proliferation was determined by sulforhodamine B assay. Data were an average of three independent experiments. Bars represent ± SD.

We examined the antiproliferative potential of 156 rhodanine compounds using HeLa cells. Among 156 compounds, three compounds, namely MNFMT, DHBPT and BCFMT (Fig. 1A), were found to inhibit HeLa cell proliferation >30% at 2 µM. MNFMT, DHBPT and BCFMT were selected for further studies. MNFMT, DHBPT and BCFMT inhibited the proliferation of HeLa (Fig. 1B) and MCF-7 (Fig. 1C) cells with a half maximal inhibitory concentration (IC_50_) of 16.8±1, 7.3±0.4, 7.2±1.8 µM, and 12.2±0.3, 4.9±0.3 and 10.0±0.5 µM, respectively.

### Effects of MNFMT, DHBPT and BCFMT on MCF-7 Cell Cycle Progression

To determine the antimitotic potential, we checked the effect of these agents on cyclin B1 which is a marker specific for G2/M phase [35]. BCFMT blocked the progression of MCF-7 cells at G2/M phase more strongly than the other two compounds (Table 1). For example, in control cells, 3.2±0.5% of the cells were positive for cyclin B1 staining whereas 11±1.5%, 9±2.5% and 29±3% cells were found to be cyclin B1 positive in the presence of 4×IC_50_ concentration of MNFMT, DHBPT and BCFMT, respectively (Table 1). In the presence of 200 and 400 nM nocodazole, 21±2% and 44±2% of cells were found to be cyclin B1 positive. We also examined the effect of these compounds on the mitotic index (number of cells in mitosis/total number of cells) of MCF-7 cells. In control, the mitotic index was found to be 3±0.5 whereas at ∼ 4×IC_50_ concentration of MNFMT (50 µM), DHBPT (20 µM) and BCFMT (40 µM) the mitotic indices were found to be 4±0.5, 6±1 and 12±1 (n = 3; in each set 1000 cells were counted), respectively. The findings together suggested that BCFMT blocked the cells in mitosis more strongly than the other two agents; therefore, we further explored the antimitotic activity of BCFMT.

**Table 1:** Effects of MNFMT, DHBPT and BCFMT on cyclin B1 expression.

Sample	% of cyclin B1 positive cells	
	2 × IC_50_	4 × IC_50_
MNFMT	6±1	11±1.5
DHBPT	5±1.5	9±2.5
BCFMT	13±1.5	29±3

In the vehicle treated (control) cells, 3.2±0.5% cells were found to be cyclin B1 positive. Data are average of 3 independent set of experiments and ± represent SD. In each set 1000 cells were counted.

### BCFMT Increased the Mitotic Index of MCF-7 and HeLa Cells

In mitotic cells histone H3 gets phosphorylated at serine 10 [36] and it is used as a marker for mitotic cells. BCFMT treatment increased the number of phosphohistone-H3 positive cells (Figure S1 in the Supporting Information). For example, 2±0.5%, 7±1%, and 11.5±1.5% cells were found to be phosphohistone-H3 positive in the absence and presence of 20 and 40 µM BCFMT, respectively. Further, BCFMT-treatment increased the metaphase/anaphase ratio in MCF-7 cells. In the vehicle-treated MCF-7 cells, the metaphase/anaphase ratio was determined to be 2.7±1.2, while in the presence of 20, 30 and 40 µM BCFMT, the metaphase/anaphase ratios were determined to be 5±1, 10±2 (p<0.001) and 15±1 (p<0.001) (n = 3; in each set 1000 cells were counted), respectively. The increased mitotic index and metaphase/anaphase ratio suggested that BCFMT inhibited the cell cycle progression of MCF-7 cells at mitosis. BCFMT also found to suppress the mitotic progression of HeLa cells as determined by mitotic index, phosphohistone-H3 staining and metaphase/anaphase ratio (Figure S2 in the Supporting Information).

Further, BCFMT treatment was found to delay the kinetics of the release of nocodazole- induced mitotic block in MCF-7 cells. For example, 60% of the cells were found to be in mitosis at the time of nocodazole washout, while 30%, 15% and 5% cells were in the mitotic phase after 1, 2 and 4 h release of the nocodazole block. In the presence of 40 µM BCFMT, 46%, 38% and 30% cells were found to be in the mitotic phase after 1, 2 and 4 h of block release suggesting that BCFMT can suppress mitotic progression.

### BCFMT Inhibited in vitro Tubulin Polymerization

**Figure 2:**
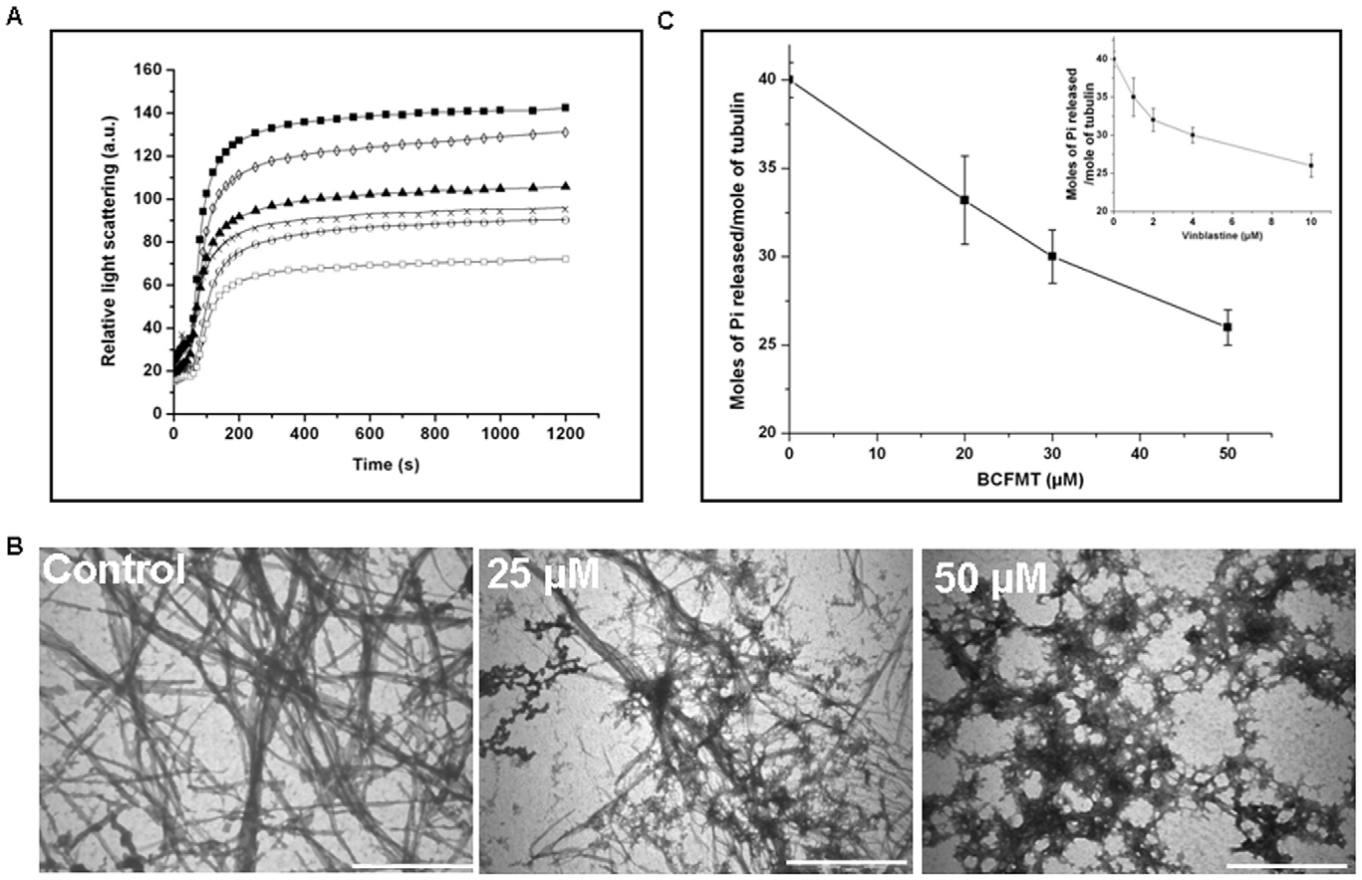
BCFMT inhibited tubulin polymerization *in vitro*. (A) Tubulin (10 µM) was polymerized in the absence (▪) and presence of 10 (◊), 25 (▴), 50 (×), 75 (○) and 100 (□) µM BCFMT. (B) Electron micrographs of tubulin polymers in the absence and presence of 25 and 50 µM BCFMT. The scale bar is 2000 nm. (C) BCFMT suppressed the GTPase activity of microtubules. Effects of vinblastine on the GTPase activity of microtubules under similar experimental conditions are shown in the inset. Data were an average of three independent experiments. Bars represent ± SD.

Since BCFMT inhibited cell cycle progression at the M phase of cell cycle; we examined whether BCFMT could perturb microtubule assembly *in vitro* and in cultured cells. BCFMT inhibited the assembly of purified tubulin in a concentration dependent manner (Fig. 2A). For example, in the presence of 25, 50 and 100 µM BCFMT, the extent of tubulin polymerization was inhibited by 27±3%, 38±4.5% and 64±3%, respectively. The initial rate of increase of the light scattering intensity of the microtubule assembly reaction was determined to be 0.97±0.03, 0.54±.07 and 0.28±0.06 (a.u./sec) in the absence and presence of 50 and 100 µM BCFMT, respectively indicating that BCFMT strongly reduced the initial rate of tubulin assembly. Electron micrographs of the control showed typical microtubule polymers (Fig. 2B). In the presence of 25 µM BCFMT, fewer microtubules were found per field of observation than the control. It also induced aggregation of tubulin dimers. In the presence of 50 µM BCFMT, microtubule formation was strongly inhibited and only tubulin aggregates were found (Fig. 2B). Under similar conditions, DHBPT and MNFMT had no significant effect on microtubule assembly. For example, 50 µM DHBPT had no detectable effect on the light scattering of microtubule assembly and 50 µM MNFMT decreased the light scattering signal of microtubule assembly only by 10%.

Further, we determined the effect of BCFMT on the GTPase activity of microtubules. BCFMT decreased the release of inorganic phosphate in a concentration dependent manner. For example, 20, 30 and 50 µM BCFMT reduced the amount of inorganic phosphate released by 17%, 25% and 32%, respectively (Fig. 2C). Under similar experimental conditions, 1, 2, 4 and 10 µM vinblastine reduced the amount of inorganic phosphate released by 12%, 20%, 25% and 35%, respectively, indicating that BCFMT inhibits the GTPase activity of microtubules like vinblastine (Fig. 2C inset).

### Characterization of the Binding of BCFMT to Tubulin

**Figure 3:**
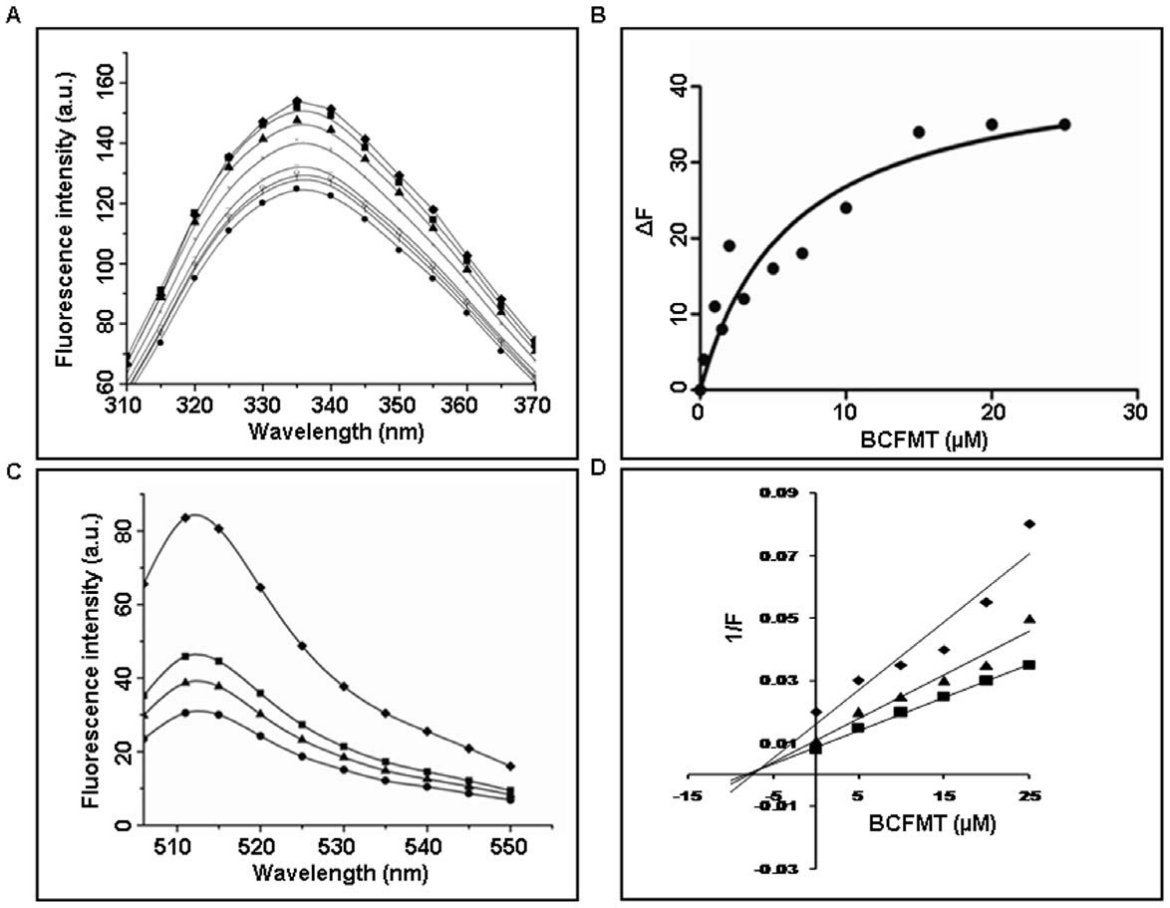
BCFMT bound to purified tubulin and inhibited the binding of BODIPY FL-vinblastine to tubulin. (A) The effects of BCFMT on the tryptophan fluorescence spectra of tubulin are shown. Spectra were monitored in the absence (♦) and presence of 0.25 (▪), 0.5 (▴), 1 (×), 2 (−), 5 (○), 7 (l) and 10 (•) µM BCFMT. (B) The change in the fluorescence intensity of tubulin (ΔF) was plotted against concentration of BCFMT. The dissociation constant (K_d_) for BCFMT binding to tubulin was estimated using an equation described in the methods. Data were the average of four independent experiments. (C) Reduction in the fluorescence intensity of tubulin- BODIPY FL-vinblastine complex in the absence (♦) and presence of 10 (▪), 25 (▴) and 50 (•) µM BCFMT. (D) Tubulin (2 µM) in 25 mM PIPES buffer (pH 6.8) was incubated without and with different concentrations (5, 10, 15, 20, 25 µM) of BCFMT at 25°C for 20 min. Three such different sets were prepared. After 20 min incubation, in one set 2 µM (♦), in the second set 4 µM (▴) and in the third set 6 µM (▪) BODIPY FL-vinblastine was added. Fluorescence of tubulin-BODIPY FL-vinblastine complex was measured and the inhibitory concentration (Ki) was calculated from the modified Dixon plot.

The intrinsic tryptophan fluorescence of tubulin has been widely used to determine the binding constant of a ligand to tubulin [37]. BCFMT reduced the intrinsic tryptophan fluorescence intensity of tubulin in a concentration dependent manner (Fig. 3A). For example, in the presence of 10 µM BCFMT tryptophan fluorescence of tubulin was reduced by 21±2.5%. Fitting the fluorescence changes in a binding isotherm yielded K_d_ of 8.3±1.8 µM (Fig. 3B).

Inhibitors of tubulin assembly generally either bind to the vinblastine or the colchicine binding sites in tubulin [11], [12]. Therefore, we examined whether BCFMT binds to tubulin at the colchicine site using colchicine-tubulin fluorescence [38]. BCFMT (10 and 20 µM) did not inhibit the development of colchicine-tubulin fluorescence indicating that it did not inhibit the binding of colchicine to tubulin (Figure S3 in the Supporting Information). BODIPY FL-vinblastine has been used to probe the binding site of vinblastine in tubulin [29]. The fluorescence intensity of BODIPY FL-vinblastine increased upon binding to tubulin (Fig. 3C). Vinblastine decreased the fluorescence enhancement of BODIPY FL-vinblastine suggesting that it binds to the vinblastine site in tubulin. BCFMT also decreased the fluorescence of BODIPY FL-vinblastine-tubulin complex in a concentration dependent fashion indicating that BCFMT inhibited the binding of BODIPY FL-vinblastine to tubulin (Fig. 3C). For example, 10, 25 and 50 µM BCFMT decreased the fluorescence of BODIPY FL-vinblastine-tubulin complex by 40±2.5%, 51±3% and 64±4%, respectively.

Further, BODIPY FL-vinblastine showed a significant increase in the fluorescence polarization value when it was bound to tubulin (Figure S4 in the Supporting Information). Inclusion of BCFMT in the reaction milieu decreased the polarization of BODIPY FL-vinblastine indicating that it reduced the binding of BODIPY FL-vinblastine to tubulin. As compared to control, 20±3%, 31±4% and 49±2% reduction in fluorescence polarization values of tubulin-BODIPY FL-vinblastine were observed in the presence of 10, 25 and 50 µM of BCFMT, respectively.

Since BCFMT inhibited BODIPY FL-vinblastine binding to tubulin, we examined the mode of inhibition using modified Dixon plot [39]. An analysis of the modified Dixon plot suggested that BCFMT inhibited the binding of BODIPY FL-vinblastine to tubulin competitively with an inhibitory concentration (K_i_) of 5.2±1.5 µM (Fig. 3D).

### A Brief Exposure of BCFMT Depolymerized Microtubules in MCF-7 Cells

**Figure 4:**
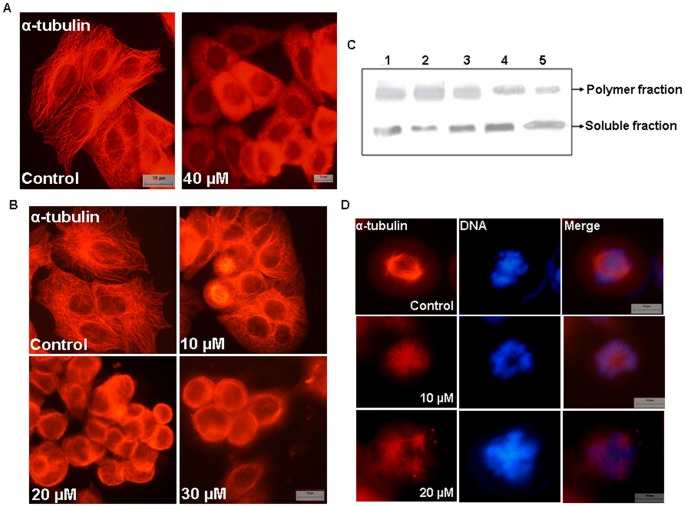
BCFMT depolymerized microtubules of MCF-7 cells. (A) Cells were treated without and with 40 µM BCFMT for 3 h and microtubules were stained using antibody against α-tubulin (red). (B) BCFMT perturbed interphase microtubule organization of MCF-7 cells. MCF-7 cells were incubated in the absence and presence of different concentrations of BCFMT for 48 h. Cells were fixed and stained using antibody against α-tubulin (red). (C) BCFMT decreased the ratio of polymeric/soluble tubulin in MCF-7 cells determined by western blot. MCF-7 cells were treated without (lane 1) or with 20 µM (lane 4) and 40 µM (lane 5) of BCFMT for 36 h. 20 nM taxol (lane 2) and 200 nM nocodazole (lane 3) were also used under similar experimental conditions. Polymeric and soluble tubulin fractions were isolated and equal amounts of proteins were loaded on SDS-PAGE. Immunoblotting was done with α-tubulin antibody. Experiment was performed independently three times. Shown is the representative blot. (D) BCFMT depolymerized spindle microtubules in MCF-7 cells. DNA stained in blue. Scale bar is 10 µm.

To examine the effect of BCFMT on cellular microtubules, MCF-7 cells were incubated without and with 40 µM BCFMT for 3 h. Vehicle-treated cells displayed typical network of microtubules while BCFMT (40 µM) treated cells showed a significant depolymerization of the microtubules (Fig. 4A). Microtubule polymers were not visible in 40 µM BCFMT-treated cells; a diffuse staining for soluble tubulin was observed in the treated MCF-7 cells.

### BCFMT Depolymerized Microtubules of MCF-7 and HeLa Cells

MCF-7 or HeLa cells were incubated with different concentrations of BCFMT for one cell cycle. BCFMT depolymerized interphase microtubules in MCF-7 cells in a concentration dependent manner. For example, the microtubule network was not visibly perturbed in the presence of 10 µM BCFMT while 20 µM BCFMT induced a significant depolymerization of the interphase microtubules and a strong depolymerization of microtubules was observed in the presence of 30 µM BCFMT (Fig. 4B). Western blot analysis indicated that in vehicle-treated MCF-7 cells, the ratio of polymeric to soluble tubulin was 2.2±0.3 whereas it was 1.2±0.1 and 0.8±0.1 in the presence of 20 and 40 µM BCFMT, respectively suggesting that BCFMT treatment depolymerized cellular microtubules (Fig. 4C). The polymer to soluble tubulin ratio in MCF-7 cells was found to be 3.1±0.2 in the presence of 20 nM taxol and 1.1±0.1 in the presence of 200 nM nocodazole (Fig. 4C). BCFMT-treatment depolymerized spindle microtubules in MCF-7 cells and also induced the formation of monopolar or multipolar spindles with misaligned chromosomes at the metaphase plate (Fig. 4D). Similar to its effects on MCF-7 cells, BCFMT depolymerized microtubules in HeLa cells in its effective proliferation inhibitory concentration range (Figure S5 in the Supporting Information). However, BCFMT (15 and 30 µM) treatment did not perturb the organization of actin fibers in MCF-7 cells (Figure S6 in the Supporting Information).

### BCFMT Suppressed Microtubule Reassembly in MCF-7 Cells

Microtubules were depolymerized by incubating MCF-7 cells on ice for 1 h and subsequently, the growth kinetics of interphase microtubules were monitored by incubating the cells at 37°C. Microtubules of the vehicle-treated cells grew rapidly and attained normal interphase network within 30 min while BCFMT (40 µM) treatment strongly suppressed the growth of microtubules (Figure S7A in the Supporting Information).

Further, the effect of BCFMT on the assembly kinetics of the spindle in mitotic MCF-7 cells was analyzed. Cells were first blocked in mitosis by incubating them with 1 µM nocodazole for 20 h. Cells were carefully washed with fresh media and further incubated on ice for 30 min without and with 40 µM BCFMT. Subsequently, cells were placed at 37°C and the reassembly kinetics of the spindle microtubules was monitored by staining the microtubules at different time intervals. In control cells, spindle microtubules polymerized rapidly and nearly normal spindles were observed within 30 min of assembly (Figure S7B in the Supporting Information, white arrow). BCFMT (40 µM) strongly suppressed the growth of spindle microtubules and the spindles did not form within 30 min of growth kinetics (Figure S7B in the Supporting Information).

### BCFMT Suppressed Dynamic Instability of Microtubules in MCF-7 Cells

**Figure 5:**
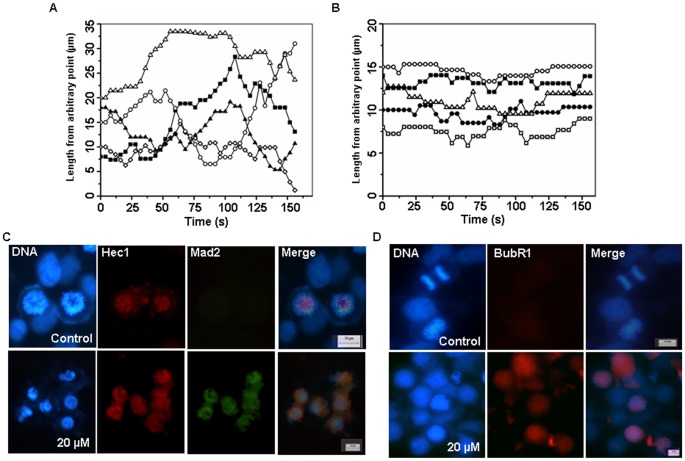
BCFMT-treatment dampened dynamics of individual microtubules and accumulated spindle assembly checkpoint proteins at the kinetochores in MCF-7 cells. (A & B) Life history traces shows microtubule length changes with time in the absence (A) and presence of 10 µM BCFMT (B). The initial length represents a length from an arbitrary fixed point of a microtubule to the plus end of the microtubule. (C & D) BCFMT treatment accumulated checkpoint proteins Mad2 (green) (C) and BubR1 (red) (D) at the kinetochores. The position of kinetochores was visualized using antibody against Hec1 (red) (C). DNA stained in blue. Scale bar is 10 µm.

As reported earlier [29], [31], [32], microtubules in the vehicle-treated MCF-7 cells showed transition between the growth, shortening and pause states and were found to be highly dynamic (Fig. 5A). BCFMT treatment reduced the dynamic instability of microtubules (Fig. 5B). For example, BCFMT (10 µM, IC_50_) decreased the rates of growing and shortening of individual microtubules by 37% and 40%, respectively (Table 2). It also reduced the mean growth and shortening lengths of microtubules by 64% and 62%, respectively. Further, it reduced the time spent in growth and shortening phases by 47% and 49% whereas increased the pause time (neither growing nor shortening detectably) by 135% as compared to control microtubules. BCFMT (10 µM) treatment increased the catastrophe (a transition from a growth or a pause state to a shortening state) and rescue (a transition from a shortening to a growth or a pause state) [31], [40] frequencies (events/µm) by 170% and 88% and decreased the dynamicity (dimer exchange per min) of microtubules by 70%.

**Table 2:** BCFMT suppressed the dynamic instability of individual microtubules in MCF-7 cells.

		Control	10 µM BCFMT
Rate (µm/min)	Growing	19.7±3.8	12.4±2†
	Shortening	21.1±3.4	12.7±2†
Length change (µm)	Growth length	2.8±1.3	1.0±0.2†
	Shortening length	2.9±1.5	1.1±0.3†
% time spent in different phases	Growth	40.0±8	21.2±5†
	Shortening	33.7±5	17.1±6†
	Pause	26.3±5	61.7±14†
Frequency (events/min)	Catastrophe	3.9±1.3	2.6±0.8†
	Rescue	7.6±1.7	11.6±2.7†
Frequency (events/µm)	Catastrophe	0.37±0.2	1.0±0.3†
	Rescue	0.50±0.7	0.94±0.3*
Dynamicity (µm/min)		14.6±2.5	4.4±1†

Data are average ± SD,

n = 25 MTs for each case.

† P<0.0001.

* P<0.001.

(Significance test was done by One-way ANOA).

### BCFMT Treatment Increased the Accumulation of Checkpoint Proteins Mad2 and BubR1 at the Kinetochores in MCF-7 Cells

BCFMT perturbed the mitotic spindle organization and induced mitotic block in MCF-7 cells. Therefore, we examined the status of spindle checkpoint proteins Mad2 and BubR1 in BCFMT-treated MCF-7 cells, which are known to get accumulated on kinetochores when microtubules are not properly attached with the kinetochores. BCFMT treatment increased the accumulation of Mad2 (Fig. 5C) and BubR1 (Fig. 5D) at kinetochores indicating that BCFMT treatment caused sustained accumulation of the mitotic checkpoint proteins leading to the mitotic arrest.

### BCFMT Caused Apoptosis in MCF-7 Cells

**Figure 6:**
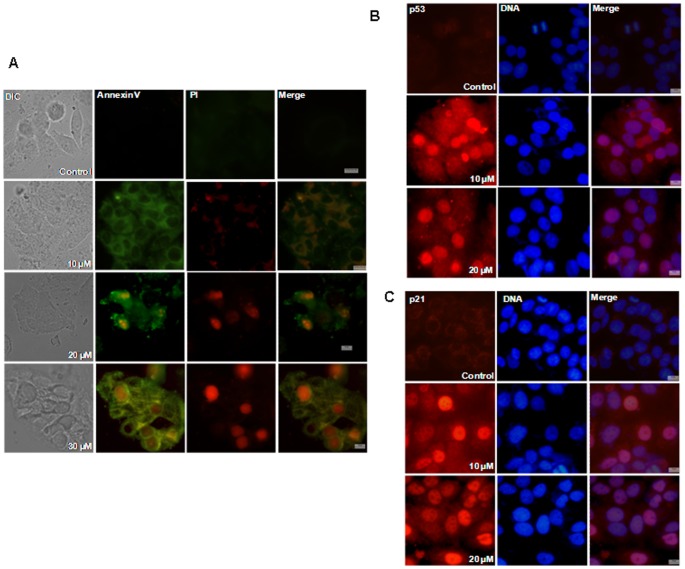
BCFMT activated p53 dependent apoptotic pathway in MCF-7 cells. MCF-7 cells were incubated without and with different concentrations of BCFMT for 48 h. (A) Cells were processed for Annexin V/PI staining. Annexin V stained cells are in green and PI stained cells are in red. (B & C) MCF-7 cells treated without or with BCFMT were fixed and stained with antibody specific for p53 (red) (B) and p21 (red) (C). DNA was stained in blue. Scale bar is 10 µm.

Differential interference contrast (DIC) images of vehicle-treated MCF-7 cells showed well defined morphology while BCFMT-treatment induced apoptotic body formation in the cells. Annexin V/PI staining clearly indicated that BCFMT treatment caused apoptosis in MCF-7 cells (Fig. 6A).

A short exposure of BCFMT also inhibited MCF-7 cells proliferation. MCF-7 cells were incubated in the absence and presence of 50 µM BCFMT or with 500 nM nocodazole for 5 h. After the incubation, BCFMT or nocodazole containing media was removed; cells were washed with fresh media and were further incubated in fresh media for 40 h. The short exposure of 50 µM BCFMT inhibited MCF-7 cell proliferation by 43%, whereas 13% inhibition was observed in the presence of 500 nM nocodazole. Further, Annexin V/PI staining indicated that a brief treatment of BCFMT caused cell death in MCF-7 cells. For example, 1%, 32%, 15% and 35% of MCF-7 cells showed PI staining in the absence and presence of 50 µM BCFMT, 500 nM nocodazole and 100 nM vinblastine, respectively.

### BCFMT Increased the Nuclear Accumulation of p53 and p21

Microtubule targeting agents are known to induce the apoptosis via activation and nuclear translocation of p53 [16], [17], [32]. BCFMT treatment increased nuclear accumulation of p53 in MCF-7 cells. For example, 1%, 20% and 55% of the MCF-7 cells were found to have nuclear accumulation of p53 in the absence and presence of 10 and 20 µM of BCFMT, respectively (Fig. 6B). Consistent with the nuclear accumulation of p53, ∼1%, 18% and 47% of the MCF-7 cells had p21 accumulation in the nucleus in the absence and presence of 10 and 20 µM of the BCFMT, respectively (Fig. 6C) indicating that BCFMT induces apoptosis in MCF-7 cells through p53 dependent pathway.

### BCFMT Inhibited Proliferation of A2780-cis, EMT6/AR1 and MDA-MB-231 Cells

To further explore the antitumor activity of BCFMT, the effects of BCFMT on the proliferation of A2780-cis (a cisplatin resistant cell line), EMT6/AR1 (a multi-drug resistant cell line known to be cross resistant against vincristine and colchicine) and MDA-MB-231 (a highly metastatic breast cancer cell line) cells were determined in culture. BCFMT inhibited the proliferation of A2780-cis, EMT6/AR1 and MDA-MB-231 cells in dose dependent manner with an IC_50_ value of 5.8±0.3, 6.5±1 µM and 6±1 µM, respectively (Figure S8 in the Supporting Information). The results suggested that BCFMT inhibited the proliferation of different types of cancer cells including drug resistant and metastatic cancer cells.

## Discussion

We identified a tubulin-targeted potential anticancer agent BCFMT from a library of rhodanine derivatives. Though two other rhodanine derivatives namely MNFMT and DHBPT also inhibited cancer cell proliferation, DHBPT did not inhibit tubulin assembly while MNFMT displayed much weaker inhibitory effect on microtubule assembly *in vitro* than that of BCFMT. These three compounds contain 2-thioxothiazolidin-4-one as a common moiety and have substitutions at different positions of the heterocyclic ring (Fig. 1A). DHBPT has substitutions at the third and fifth positions whereas MNFMT and BCFMT have modification only at the fifth position. DHBPT did not affect tubulin assembly suggesting that the dual substitutions in the rhodanine scaffold might be inhibiting its ability to interact with tubulin. BCFMT has two halogen atoms, bromine and chlorine while MNFMT has a nitro and a methyl group substitution. It has been suggested that halogen atoms in ligand may increase the binding affinity of the ligand to protein and can also enhance the stability of the ligand-protein complex by forming halogen bonds with the protein [41], [42]. Thus, the presence of chlorine and bromine might be involved in the formation of halogen bonds with tubulin rendering BCFMT more active than MNFMT.

In its effective proliferation inhibitory concentration range, BCFMT depolymerized microtubules of MCF-7 and HeLa cells in culture, strongly suppressed the dynamic instability of microtubules in live MCF-7 cells, bound to purified tubulin, inhibited reconstituted microtubule assembly and suppressed the GTPase activity of microtubules *in vitro*. In addition, BCFMT disrupted the mitotic spindles, misaligned chromosomes, increased the number of cell in the G2/M phase of cell cycle and activated the mitotic checkpoint proteins BubR1 and Mad2 suggesting that it inhibits cell proliferation by inhibiting microtubule assembly dynamics. Further, a brief exposure with BCFMT caused significant depolymerization of cellular microtubules, inhibited cell proliferation and induced cell death in MCF-7 cells suggesting that a short exposure of BCFMT is able to cause irreversible microtubule damage in the cells.

BCFMT inhibited the binding of BODIPY FL-vinblastine to tubulin and an analysis of the modified Dixon plot suggested that BCFMT competitively inhibits BODIPY FL-vinblastine binding to tubulin with a Ki value of 5.2 µM indicating that BCFMT binds to tubulin at the vinblastine site (Fig. 3D). Several of the clinically successful tubulin-targeted anticancer agents such as vincristine, vinorelbine, vinflunine bind to the vinblastine site in tubulin. In addition, several other vinca binding agents like dolastatins, cryptophycin 52, maytansine and its derivatives, and hemiasterlins have either been tested or are under various phases of clinical trials suggesting that the vinca binding agents play an important role in cancer chemotherapy [11]. Since BCFMT binds to the vinblastine binding site in tubulin and inhibits cancer cell proliferation by targeting microtubule assembly, it may also have anticancer potential. The antiproliferative activity of BCFMT is much weaker than that of vinblastine. Therefore, BCFMT may be used as a lead compound to develop potent tubulin-targeted rhodanine derivatives.

Vinblastine, cryptophycin 52, maytansine, phomopsin A have complex closed ring conformation whereas dolastatin 10, dolastatin 15, hemiasterlins, soblidotin are long straight chain compounds revealing that the vinca domain binding agents are complex and diverse in structures. Interestingly, BCFMT does not share an apparent structural similarity with the known vinca- binding agents indicating a new chemical entity; thus, it may have anticancer potential particularly in combination with other agents.

BCFMT bound to tubulin with a K_d_ of 8.3±1.8 µM. As compared to other known microtubule depolymerizing agents like vinblastine (K_d_ = 43 µM) [43], dolastatin 15 (K_d_ = 30 µM) [44] and estramustine (K_d_ = 30 µM) [9], BCFMT was found to interact strongly with tubulin. However, it showed lower affinity for tubulin as compared to colchicine (K_d_ = 0.5 µM) [45] and cryptophycin-5 (K_d_ = 47 nM) [46]. BCFMT inhibited HeLa, MCF-7, MDA-MB-231 A2780-cis and multi-drug resistant EMT6/AR1 cell proliferation with IC_50_ values of 7.2±1.8 µM, 10.0±0.5 µM, 6±1 µM, 5.8±0.3 µM and 6.5±1.0 µM, respectively. The IC_50_ values of BCFMT are comparable with that of several other known tubulin targeting agents like estramustine (5 µM) [31], griseofulvin (17 µM) [29], benomyl (5 µM) [47] and noscapine (33.4 µM) [48].

Like several other microtubule targeting anticancer agents [11], [12], BCFMT also suppressed microtubule dynamics without visibly perturbing the microtubule network in cultured cells. At half maximal inhibitory concentration, BCFMT decreased the shortening rate of individual microtubules by 40% in MCF-7 cells while vinblastine decreased the shortening rate of microtubules by 30% in BS-C-1 cells [8]. However, BCFMT displayed more pronounced effect on the growth rate of microtubules than that of vinblastine [8]. Similar to the effects of vinblastine on the transition frequencies of microtubules, BCFMT also decreased the catastrophe frequency and increased the rescue frequency of microtubules. At half maximal inhibitory concentrations, BCFMT suppressed the dynamicity of microtubules by ∼70%, while under similar proliferation inhibitory conditions; vinblastine suppressed the dynamicity of microtubules by 44% [8]. The analogous effects of BCFMT and vinblastine on the different parameters of dynamic instability suggested that BCFMT and vinblastine exerted similar actions on microtubule dynamics. Dynamic microtubules are important for the formation of proper bipolar spindle. It has been suggested that the dampening of microtubule dynamics led to the accumulation of spindle checkpoint proteins at the kinetochores in response to less tension and improper microtubule-kinetochore attachments [29], [31], [49]. Biochemical (cyclin B1 and phosphohistone-H3 staining), phenotypic (Hoechst 33258 staining) and kinetic experiments suggested BCFMT slows mitosis in cultured cells. In BCFMT-treated cells, microtubules were not correctly attached to the kinetochores that delayed the mitotic progression with the accumulation of checkpoint proteins Mad2 and BubR1.

In some instances, microtubule targeting agents such as paclitaxel and vinflunine have been found to induce cell death without arresting cells in mitosis [50]–[52]. For example, vinflunine caused apoptosis but was unable to inhibit human SK-N-SH neuroblastoma cells progression in G2/M phase even at concentrations where it inhibits 50 to 70% cell proliferation [50] or paclitaxel at its low effective concentration has been found to induce cell death without a prior G2/M arrest [51], [52]. Low concentrations of vinflunine and paclitaxel are known to suppress microtubule dynamics without significantly altering the microtubule network in the cells [11], [50], [53]. It is possible that the suppression of microtubule dynamics by BCFMT also activates the cell death program before the treated cells enter into mitosis; therefore, a strong mitotic block has not been observed.

BCFMT treatment induced apoptotic death in HeLa and MCF-7 cells. Microtubule targeting agents are shown to induce apoptosis by different pathways [16], [17]. Microtubule inhibitors are known to increase the expression and nuclear translocation of p53 and p21 [32], [54]. In MCF-7 cells BCFMT treatment caused apoptosis via activation and nuclear translocation of p53 and its downstream protein p21 suggesting that BCFMT can induce apoptosis through p53 dependent pathway.

The current limitations of the existing anticancer drugs include the development of resistance against them and tumor metastasis [1], [2]. BCFMT inhibited the proliferation of highly metastatic MDA-MB-231 cells, multi-drug resistant EMT6/AR1 cells that display cross resistance to tubulin targeting agents like vincristine and colchicine and cisplatin-resistant A2780-cis cells. This suggests that BCFMT has potential to overcome the problem of drug resistance and might find utility in case of tumors, which are resistant to commonly used tubulin-targeting agents.

In conclusion, we identified a novel anti-tubulin agent BCFMT that inhibits proliferation of several types of cancer cells including drug resistance cells by suppressing microtubule dynamics and the results indicated that the compound may have chemotherapeutic potential.

## Supporting Information

Figure S1 **BCFMT treatment increased the number of phosphohistone positive MCF-7 cells.** MCF-7 cells were incubated without and with different concentrations of BCFMT for 48 h. Cells were fixed and then, immunostained using antibody against phosphohistone. Phosphohistone positive cells were counted. Data were an average of three independent experiments. In each set 1000 cells were counted. Bars represent ± SD.
(TIF)

Figure S2 **BCFMT suppressed the mitotic progression in HeLa cells.** HeLa cells were incubated without and with different concentrations of BCFMT for 24 h. (A) Cells were stained with Hoechst 33258 and mitotic indices were counted in the absence and presence of different concentrations of BCFMT. (B) Cells were fixed and then, immunostained using antibody against phosphohistone. Phosphohistone positive cells were visually counted. (C) Metaphase/anaphase ratio was calculated in the absence and presence of 10 and 20 µM of BCFMT. Data were an average of three independent experiments. In each set 1000 cells were counted. Bars represent ± SD.
(TIF)

Figure S3 **BCFMT did not inhibit the binding of colchicine to tubulin.** Tubulin (5 µM) in 25 mM PIPES buffer pH 6.8 was incubated without (♦) and with 10 (▪) and 20 (▴) µM of BCFMT for 20 min at 25°C. Colchicine (10 µM) was added to the reaction milieu and samples were incubated for an additional 1 h at 37°C. Fluorescence of tubulin-colchicine complex was examined by exciting the samples at 350 nm and emission was recorded in the range of 390–470 nm.
(TIF)

Figure S4 **BCFMT reduced the polarization of tubulin-BODIPY FL-vinblastine complex.** Tubulin (4 µM) in 25 mM PIPES buffer (pH 6.8) was incubated without and with different concentrations (10, 25 and 50 µM) of BCFMT at 25°C for 20 min. BODIPY FL-vinblastine (2 µM) was added in the reaction mixtures and incubated at 25 °C for an additional 20 min in dark. Polarization of tubulin-BODIPY FL-vinblastine complex at 515 nm in the absence and presence of BCFMT was measured using JASCO FDP 200/210 polarization accessories in a FP-6500 spectrofluorometer. Polarization was calculated using the equation P = (I**_vv_**–GI**_vh_**)/(I**_vv_** + GI**_vh_**) h and v represent horizontal and vertical positioning of the excitation and emission polarizer, respectively. G is the correction factor for the transmission efficiency and calculated by I**_hv_**/I**_hh_**. Data were an average of three independent experiments. Bars represent ± SD.
(TIF)

Figure S5 **BCFMT depolymerized interphase microtubules in HeLa cells.** HeLa cells were incubated without and with different concentrations of BCFMT for 24 h. Cells were fixed and then, immunostained using antibody against α-tubulin (red). DNA stained in blue. Scale bar is 10 µm.
(TIF)

Figure S6 **BCFMT did not perturb the organization of actin network in MCF-7 cells.** MCF-7 cells were incubated without and with 15 and 30 µM of BCFMT for 40 h. Cells were fixed and immunostaining was performed using antibody against β-actin (red). Scale bar is 10 µm.
(TIF)

Figure S7 **BCFMT suppressed the growth of interphase and mitotic microtubule.** (A) MCF-7 cells (8**×**10^4^ cells/ml) were incubated without and with 40 µM BCFMT on ice for 1 h. Subsequently, cells were incubated in a CO_2_ incubator at 37°C for different durations and fixed with 3.7% formaldehyde. Upper panel and lower panel show the kinetics of microtubule growth in control and 40 µM BCFMT treated MCF-7 cells, respectively. (B) MCF-7 cells were synchronized in mitosis by incubating the cells with 1 µM nocodazole for 24 h. Nocodazole containing media was removed; cells were carefully washed 4 times with fresh media and further incubated on ice for 30 min without and with 40 µM BCFMT. Cells were incubated for different durations at 37°C and fixed with 3.7% formaldehyde. Upper panel and lower panel show the assembly kinetics of spindle microtubules in the absence and presence of 40 µM BCFMT, respectively. Scale bar is 10 µm.
(TIF)

Figure S8 **BCFMT inhibited the proliferation of cisplatin-resistant A2780-cis, multi-drug resistant EMT6/AR1 and MDA-MB-231 cells.** Cells were incubated without and with different concentrations of BCFMT for 24 hours. The inhibitory effect of BCFMT on the proliferation of A2780-cis (▴), EMT6/AR1 (•) and MDA-MB-231 (□) cells was determined by sulforhodamine B assay. Data were an average of three independent experiments. The bars represent ± SD.
(TIF)
