# Supplementary figures and images for: An Antitubulin Agent BCFMT Inhibits Proliferation of Cancer Cells and Induces Cell Death by Inhibiting Microtubule Dynamics
Source: PLoS One. 2012 Aug 31;7(8):e44311. doi: 10.1371/journal.pone.0044311 (PMC3432122; doi:10.1371/journal.pone.0044311)

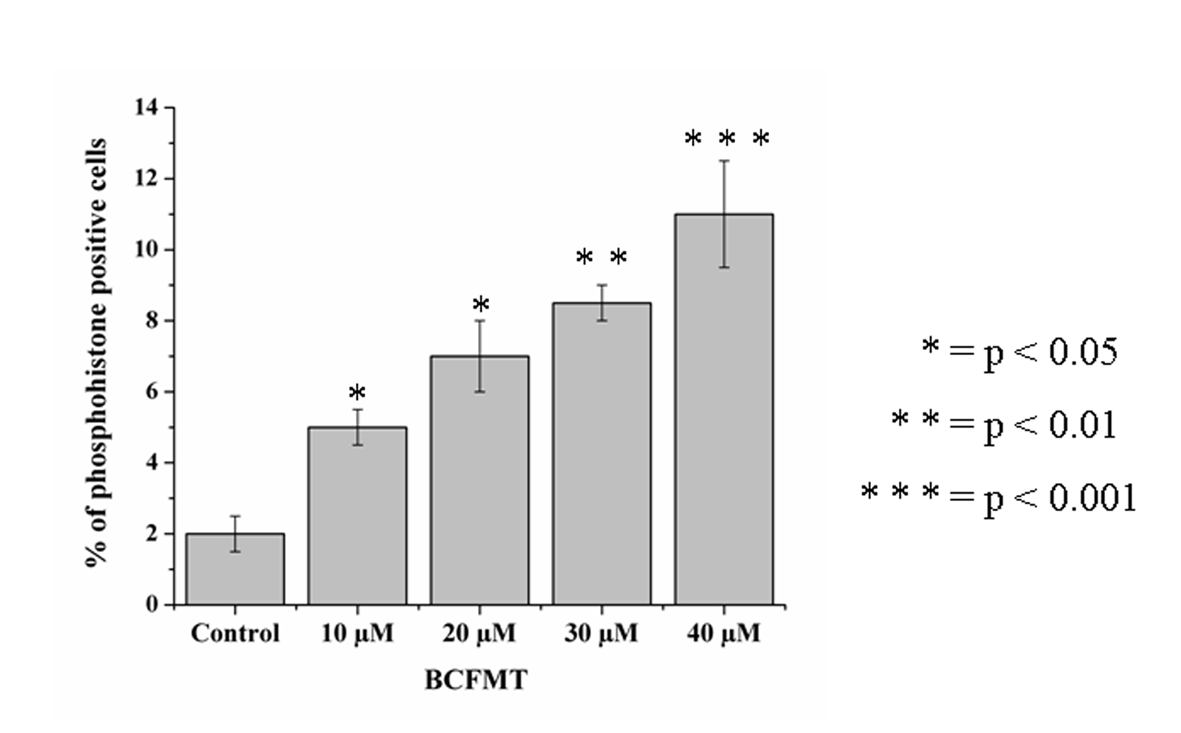

Supplement: Figure S1 — BCFMT treatment increased the number of phosphohistone positive MCF-7 cells. MCF-7 cells were incubated without and with different concentrations of BCFMT for 48 h. Cells were fixed and then, immunostained using antibody against phosphohistone. Phosphohistone positive cells were counted. Data were an average of three independent experiments. In each set 1000 cells were counted. Bars represent ± SD. (TIF) [file pone.0044311.s001.tif]

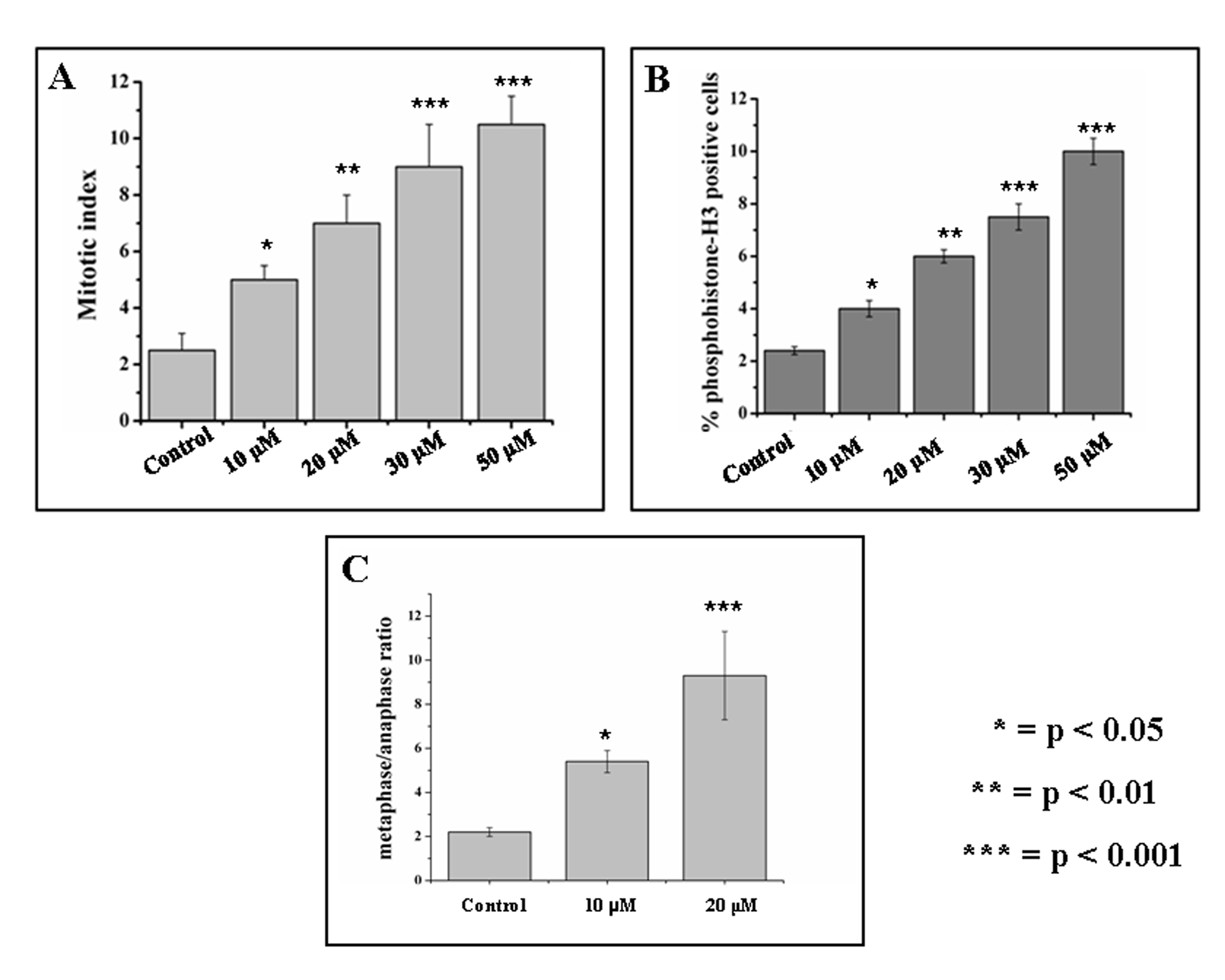

Supplement: Figure S2 — BCFMT suppressed the mitotic progression in HeLa cells. HeLa cells were incubated without and with different concentrations of BCFMT for 24 h. (A) Cells were stained with Hoechst 33258 and mitotic indices were counted in the absence and presence of different concentrations of BCFMT. (B) Cells were fixed and then, immunostained using antibody against phosphohistone. Phosphohistone positive cells were visually counted. (C) Metaphase/anaphase ratio was calculated in the absence and presence of 10 and 20 µM of BCFMT. Data were an average of three independent experiments. In each set 1000 cells were counted. Bars represent ± SD. (TIF) [file pone.0044311.s002.tif]

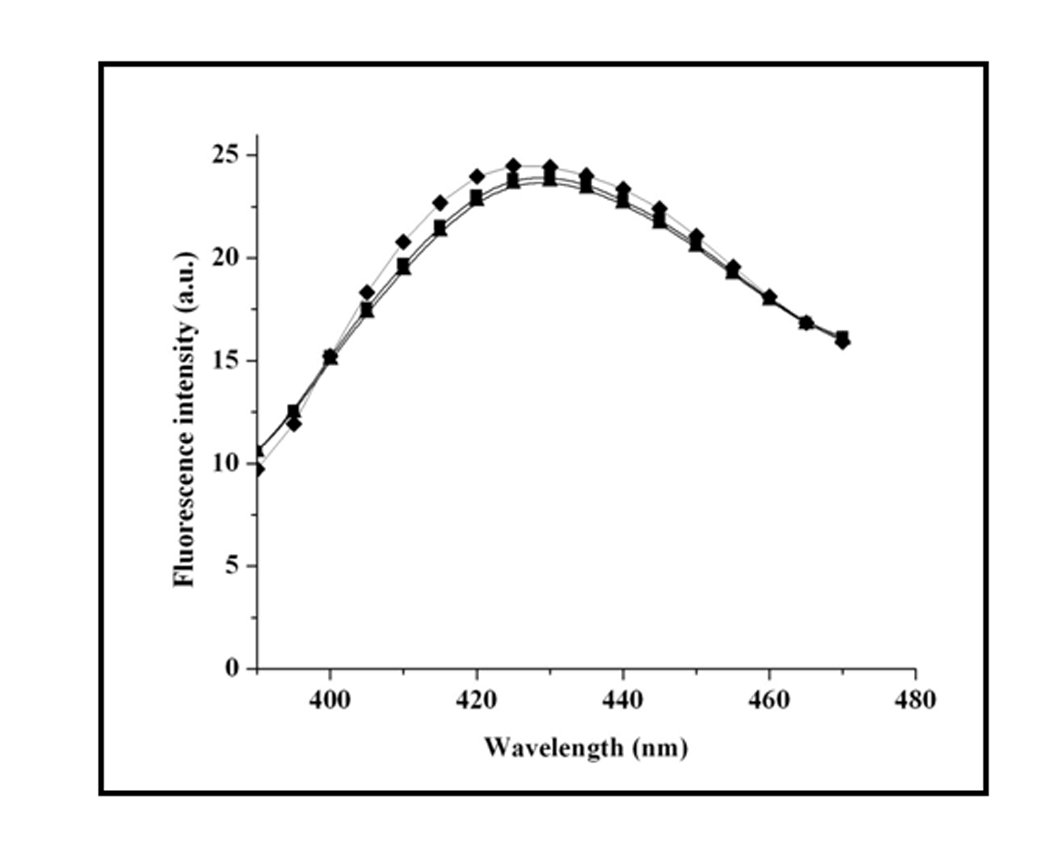

Supplement: Figure S3 — BCFMT did not inhibit the binding of colchicine to tubulin. Tubulin (5 µM) in 25 mM PIPES buffer pH 6.8 was incubated without (♦) and with 10 (▪) and 20 (▴) µM of BCFMT for 20 min at 25°C. Colchicine (10 µM) was added to the reaction milieu and samples were incubated for an additional 1 h at 37°C. Fluorescence of tubulin-colchicine complex was examined by exciting the samples at 350 nm and emission was recorded in the range of 390–470 nm. (TIF) [file pone.0044311.s003.tif]

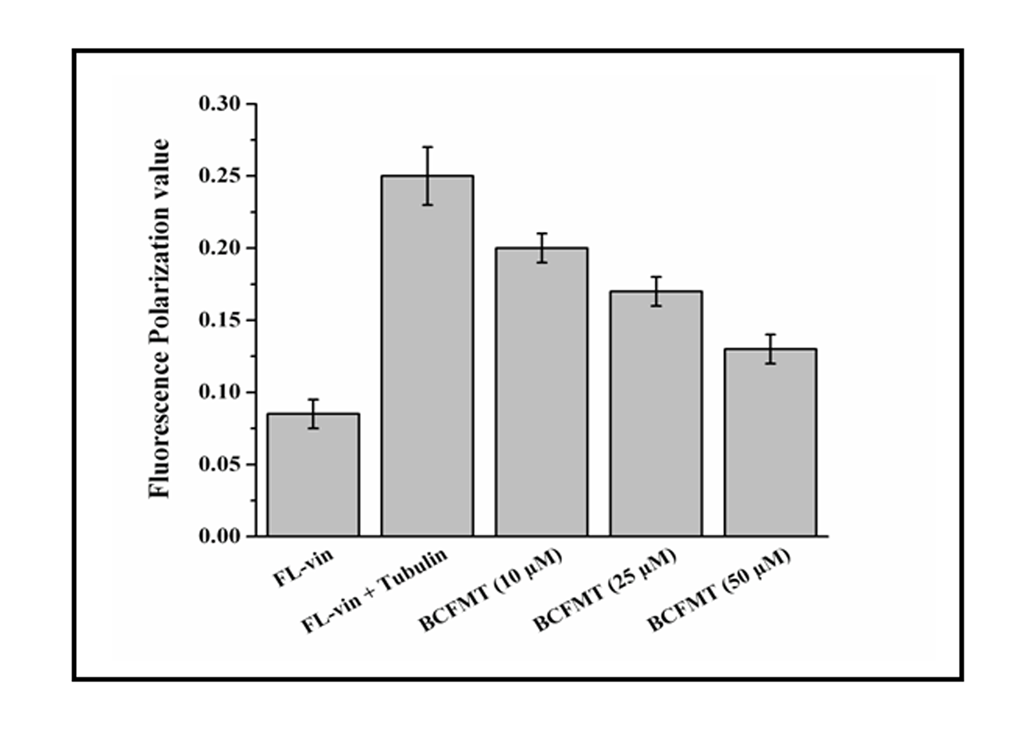

Supplement: Figure S4 — BCFMT reduced the polarization of tubulin-BODIPY FL-vinblastine complex. Tubulin (4 µM) in 25 mM PIPES buffer (pH 6.8) was incubated without and with different concentrations (10, 25 and 50 µM) of BCFMT at 25°C for 20 min. BODIPY FL-vinblastine (2 µM) was added in the reaction mixtures and incubated at 25 °C for an additional 20 min in dark. Polarization of tubulin-BODIPY FL-vinblastine complex at 515 nm in the absence and presence of BCFMT was measured using JASCO FDP 200/210 polarization accessories in a FP-6500 spectrofluorometer. Polarization was calculated using the equation P = (Ivv–GIvh)/(Ivv + GIvh) h and v represent horizontal and vertical positioning of the excitation and emission polarizer, respectively. G is the correction factor for the transmission efficiency and calculated by Ihv/Ihh. Data were an average of three independent experiments. Bars represent ± SD. (TIF) [file pone.0044311.s004.tif]

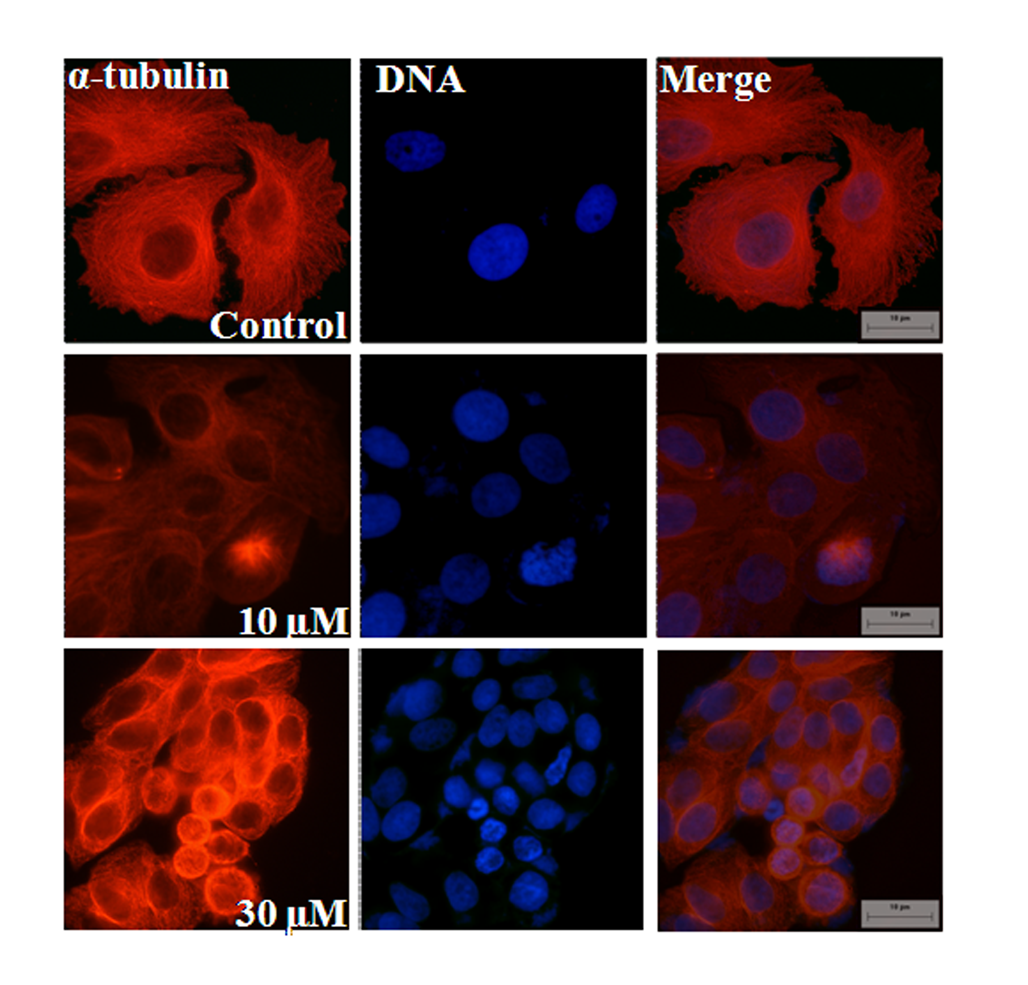

Supplement: Figure S5 — BCFMT depolymerized interphase microtubules in HeLa cells. HeLa cells were incubated without and with different concentrations of BCFMT for 24 h. Cells were fixed and then, immunostained using antibody against α-tubulin (red). DNA stained in blue. Scale bar is 10 µm. (TIF) [file pone.0044311.s005.tif]

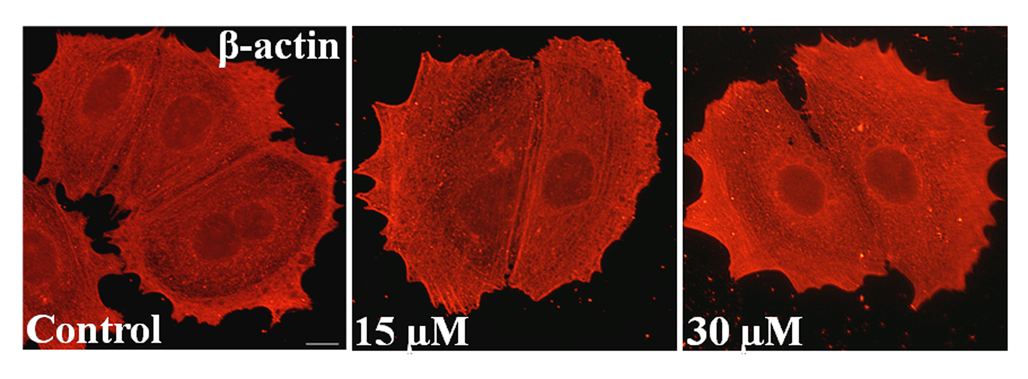

Supplement: Figure S6 — BCFMT did not perturb the organization of actin network in MCF-7 cells. MCF-7 cells were incubated without and with 15 and 30 µM of BCFMT for 40 h. Cells were fixed and immunostaining was performed using antibody against β-actin (red). Scale bar is 10 µm. (TIF) [file pone.0044311.s006.tif]

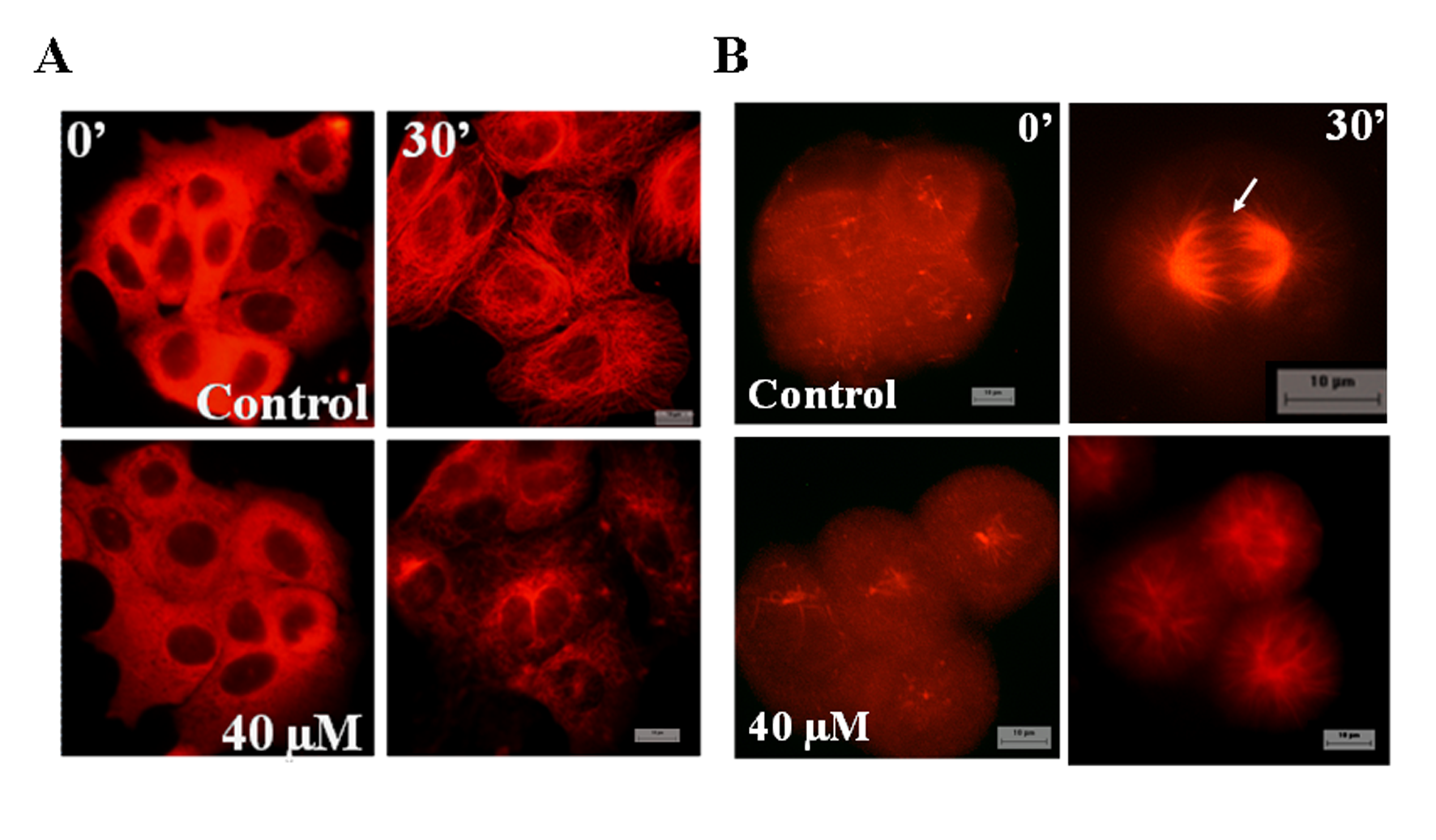

Supplement: Figure S7 — BCFMT suppressed the growth of interphase and mitotic microtubule. (A) MCF-7 cells (8×10^4^ cells/ml) were incubated without and with 40 µM BCFMT on ice for 1 h. Subsequently, cells were incubated in a CO2 incubator at 37°C for different durations and fixed with 3.7% formaldehyde. Upper panel and lower panel show the kinetics of microtubule growth in control and 40 µM BCFMT treated MCF-7 cells, respectively. (B) MCF-7 cells were synchronized in mitosis by incubating the cells with 1 µM nocodazole for 24 h. Nocodazole containing media was removed; cells were carefully washed 4 times with fresh media and further incubated on ice for 30 min without and with 40 µM BCFMT. Cells were incubated for different durations at 37°C and fixed with 3.7% formaldehyde. Upper panel and lower panel show the assembly kinetics of spindle microtubules in the absence and presence of 40 µM BCFMT, respectively. Scale bar is 10 µm. (TIF) [file pone.0044311.s007.tif]

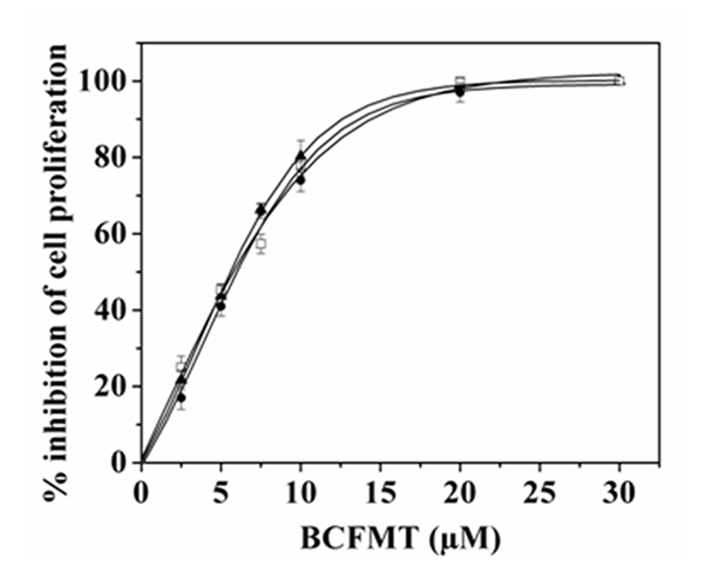

Supplement: Figure S8 — BCFMT inhibited the proliferation of cisplatin-resistant A2780-cis, multi-drug resistant EMT6/AR1 and MDA-MB-231 cells. Cells were incubated without and with different concentrations of BCFMT for 24 hours. The inhibitory effect of BCFMT on the proliferation of A2780-cis (▴), EMT6/AR1 (•) and MDA-MB-231 (□) cells was determined by sulforhodamine B assay. Data were an average of three independent experiments. The bars represent ± SD. (TIF) [file pone.0044311.s008.tif]
